# Development of a Digital Assistant to Support Teleconsultations Between Remote Physicians and Frontline Health Workers in India: User-Centered Design Approach

**DOI:** 10.2196/25361

**Published:** 2023-02-02

**Authors:** Neha Verma, Harold Lehmann, Amal Afroz Alam, Youseph Yazdi, Soumyadipta Acharya

**Affiliations:** 1 Division of Health Sciences Informatics Johns Hopkins University Baltimore, MD United States; 2 Intelehealth Baltimore, MD United States

**Keywords:** telemedicine, telehealth, eHealth, mobile health, mHealth, community health workers, frontline health workers, digital health assistant, task shifting

## Abstract

**Background:**

Many low- and middle-income countries have adopted telemedicine programs that connect frontline health workers (FHWs) such as nurses, midwives, or community health workers in rural and remote areas with physicians in urban areas to deliver care to patients. By leveraging technology to reduce temporal, financial, and geographical barriers, these health worker–to-physician telemedicine programs have the potential to increase health care quality, expand the specialties available to patients, and reduce the time and cost required to deliver care.

**Objective:**

We aimed to identify, validate, and prioritize unmet needs in the health care space of health worker–to-physician telemedicine programs and develop and refine a solution that addresses those needs.

**Methods:**

We collected information regarding user needs through ethnographic research, direct observation, and semistructured interviews with 37 stakeholders (n=5, 14% physicians; n=1, 3% public health program manager; n=12, 32% community health workers; and n=19, 51% patients) at 2 telemedicine clinics in rural West Bengal, India. We used the *Spiral-Iterative Innovation Model* to design and develop a prototype solution to meet these needs.

**Results:**

We identified 74 unmet needs through our immersion in health worker–to-physician telemedicine programs. We identified a critical unmet need that achieving optimal teleconsultations in low- and middle-income countries often requires shifting tasks such as history taking and physical examination from high-skilled remote physicians to FHWs. To meet this need, we developed a prototype digital assistant that would allow FHWs to assume some of the tasks carried out by remote clinicians. The user needs of multiple stakeholder groups (patients, FHWs, physicians, and health organizations) were incorporated into the design and features of the task-shifting tool. The final prototype was shared with the health workers, physicians, and public health program managers who expressed that the tool would be useful and valuable.

**Conclusions:**

The final prototype that was developed was released as an open-source digital public good and may improve the quality and efficiency of care delivery in health worker–to-physician telemedicine programs.

## Introduction

### Background

Over 3.8 billion people, half the world’s population, lack access to essential medical care globally [1]. In low- and middle-income countries (LMICs) such as India, people living in rural areas must often travel far and spend a significant amount of time and money to access even basic medical care [2]. Despite the cost, they face long wait times and suboptimal quality of care at overburdened government facilities or have to navigate a fragmented private sector resulting in high out-of-pocket expenditures [2]. Several telemedicine initiatives have been implemented by different organizations in India [3-6] with unique considerations and implications for health care delivery. Prominent among these are the health worker–to-physician programs, which are defined as those where frontline health workers (FHWs) can facilitate teleconsultations for a patient with a licensed physician [7]. During teleconsultation, the health worker can take the history, examine the patient, and convey the findings to the physician [7]. They can explain and reiterate the physician’s advice to the patient [7]. The term FHW encompasses different cadres of health workers, including pharmacists, nurses, and volunteer community health workers [8], with each group having varying levels of training and experience [8]. The use of technology and mobile tools to support FHWs at the point of care is well studied in the literature for various use cases such as community-based information systems, electronic medical records (EMRs), learning and training systems, and telemedicine [8-10]

Through clinical immersion at 2 telemedicine clinics in 1 such project (Rural Health Kiosk, launched in 2015) in rural and remote areas in West Bengal, India, we studied the process of care delivery in health worker–to-physician telemedicine programs. The Rural Health Kiosk project aimed to reduce the challenges of geographical access to health care in the hinterlands of West Bengal. Two teleclinics were set up under this project in Barhra, a remote village, and Bali, an island in the Sundarban delta. These clinics are operated by local females from the village (called health assistants [HAs]) who have completed a government-accredited paramedical training program. HAs connect patients from the village to backend physicians who are based in Kolkata city using low bandwidth telemedicine technology for evaluation and decision-making in the clinical management pathway. They also conduct some diagnostic tests using low-cost point-of-care diagnostic sensors (eg, electrocardiogram and blood glucose). The physician communicates with the HA and the patient and accordingly prescribes medications and advice. This information is then transmitted to the HA who prints the prescription and explains and hands it over to the patient. Patients can purchase medicines via a pharmacy near the kiosk. In addition, the physician visits the kiosk once a month to administer care to patients that cannot be managed via telemedicine. Emergency patients are referred to the nearest secondary or tertiary care facility. Patients pay a small fee for the services, and additional revenue streams are generated through the sale of medicine, physiotherapy, and diagnostic pathology tests. The HA also provides community health care services such as immunization awareness, ante- and postnatal care, newborn care, and adolescent reproductive health awareness.

In a prior publication, we described the development of a software platform to support teleconsultations between FHWs and remote physicians [11]. In this paper, we describe the design and development of a key component of the software platform, a digital assistant to task shift history taking and physical examination to FHWs so that the remote physician has some initial information before beginning the teleconsultation. The use of digital assistants to improve the quality of clinical information gathered in a health care setting has been well studied in high-resource country settings [12-15]. We hypothesized that such a digital assistant could improve the quality of clinical information gathered in the teleconsultation process. It would have an added significance in an LMIC setting where physicians have to deal with high patient loads [16] and where diagnostic testing access is poor, leading to a greater reliance of physicians on the patient history to make a diagnosis [17].

### Objectives

The objectives of this study were as follows: (1) to understand the needs of all stakeholders (patients, FHWs, physicians, and health organizations) during a telemedicine encounter between a rural patient and remote physician, which is facilitated by a health worker, and (2) to develop a solution to meet these needs. We used a bedside-to-bench-to-bedside approach from the *Spiral-Iterative Innovation Model* for bioengineering innovation and design [18]. Many digital health projects do not move beyond the pilot stage, an important reason being that the needs and wants of stakeholders are not taken into account or that the system is not well designed for a local context [18-21]. Hence, we used a human-centered design approach and clinical immersion in the project location to incorporate the perspectives of stakeholders into the design of a solution and mitigate the risks of poor user adoption. We used the *Spiral-Iterative Innovation Model* [18] a biodesign approach to understanding, validating, and prioritizing unmet user needs in the health care space, as well as the creation, assessment, and refinement of solutions that address those needs (Multimedia Appendix 1 provides a detailed diagram of the model). The model requires designers to consider the perspectives and needs of all people and organizations impacted by the problem and its solution. These perspectives and their issues are organized into 4 domains—“clinical/medical, commercial/business, technical/design, and strategic/organizational”—that are evaluated during multiple iterations of the design process [22].

This paper describes the observations, insights, and unmet user needs that we identified. On the basis of these needs, we developed a prototype task-shifting digital assistant to support teleconsultations between FHWs and physicians, which was validated through user feedback. We believe that the findings of this study are highly relevant because access to qualified health care providers is a major challenge in rural areas, which has only been exacerbated by the COVID-19 pandemic. Travel restrictions, rising unemployment, and fear of visiting health facilities further prevent rural patients from seeking care when and where they need it, lending greater importance to telemedicine.

## Methods

We performed ethnographic research by direct observation and through key stakeholder interviews between August 2015 and June 2016 at 2 teleclinics of the “Rural Health Kiosk project” in West Bengal, India, implemented by JSV Innovations.

### Design Methods: The Spiral-Iterative Innovation Model

We performed 3 design iterations using this model that are listed in subsequent sections.

#### Opportunity Discovery

The first step in the Spiral-Iterative Innovation Model process is the careful observation and extraction of actionable insights into potential stakeholders’ needs in the health worker–to-physician telemedicine encounter.

A team of 6 researchers (graduate students) performed the ethnographic research and shadowed health workers, physicians, and patients at various locations. We observed a total of 37 stakeholders (n=5, 14% physicians; n=1, 3% public health program manager; n=12, 32% community health workers; and n=19, 51% patients) during the opportunity discovery phase at various clinical sites. Clinical immersions were carried out at the following 5 locations: 2 teleclinics, 1 hub hospital, and 2 physician’s homes. Clinical sites were chosen to include all the possible locations involved in a health worker–to-physician telemedicine encounter (maximum variation sampling). We surveyed all health workers, physicians, and program managers involved with the Rural Health Kiosk project. The 12 health workers (6 at each teleclinic location) were females between the ages of 20 and 50 years. They lived in the communities served by these clinics. Their education level ranged from class 8 to graduate level. All health workers were certified “Home Health Aides,” a government-recognized allied health and paramedical training certificate program. Remote physicians were general physicians with an MD degree and between 20 and 40 years of clinical experience. The limited number of health workers and physicians would impact the generalizability of results; hence, we tried to limit our needs to those expressed during the health worker–patient-physician teleconsultation and not individually expressed opinions.

We used a purposive sampling approach for patients. The health workers and program manager invited community members to participate in an observed teleconsultation. The community members were invited such that their characteristics represented the types of patients who visit the teleclinic, including adult male and female patients (aged 18-65 years); children and adolescents (aged 0-18 years); older adults (aged ≥65 years); and patients who were living below the poverty line; had low literacy; belonged to scheduled castes, scheduled tribes, or other backward castes; and were farmers. We stopped recruiting more patients when saturation was reached, and very little new information was gained by the observation of a patient encounter.

We obtained oral consent from stakeholders before the interview or observation of the patient visit. The interviews were conducted in Bengali, Hindi, or English and were held at the clinical immersion site soon after the telemedicine encounter. They were individual interviews lasting between 30 and 60 minutes. The program manager (who was familiar with all 3 languages) served as a translator and was present for the observations and interviews. In addition, 2 members of the study team were fluent in Bengali and 2 in Hindi.

Observations were made through immersion into the clinical environment to study the behaviors, perspectives, and challenges of the stakeholders and factors in the user environment that influence and shape stakeholder behavior [18]. To ensure the completeness of capture of these observations, we sought multiple field settings, including rural telemedicine clinics, hub hospitals, and homes of remote physicians who would respond to cases while working from home. The observations were recorded with an observation code, date, location, persons involved, and a brief description of the activity being observed. After the teleconsultation, we conducted semistructured interviews with the patient, health worker, physician, and program manager to understand the barriers and facilitators that they experienced in the teleconsultation process.

#### Needs Selection

The unmet needs identified through direct observation and interviews were analyzed using root cause analysis to arrive at the unmet user need. These were developed into unique “needs statements” using a standardized format consisting of a subject, a verb, a desired outcome, and optionally additional context. For example, “A doctor (subject) needs a way to remotely collect (verb) a patient’s clinical information (desired outcome) in order to provide an accurate diagnosis and treatment plan (context)” [22,23]. Each needs statement was provided a code number and linked with the observations from which it was derived. Issues from each of the 4 quadrants were considered from each stakeholder’s perspective. Using the 4 domains, we clustered user needs into thematic areas, filtered them, and prioritized them to select the most critical need to be addressed.

#### Solution Design

We developed a prototype of the solution to meet the need informed by all 4 perspectives. The prototype solution was shared with the users who were observed to understand if the design was acceptable and met their needs. This was done through user feedback interviews with the community health workers (2 group settings with 6 community health workers in each group) and individual interviews with each of the physicians and the public health program manager. The feedback from the participants was analyzed and incorporated into the final design of the solution.

### Ethical Considerations

This study was approved by the Johns Hopkins Institutional Review Board and the JSV Innovations Institutional Review Board in India, and informed consent was obtained as per protocol (IRB00050927).

## Results and Discussion

In this paper, we present a combined Results and Discussion section because of the iterative nature of the design method used. The results and the corresponding discussion of the significance of these results for each design iteration are presented in subsequent sections.

### Iteration 1: Opportunity Discovery

We identified 74 unmet needs at various stages of the teleconsultation workflow, which were developed into needs statements. These needs have been clustered into 11 thematic areas and presented along with example needs statements in Table 1. Multimedia Appendix 2 provides the complete list of needs statements.

**Table 1:** Selected needs statements identified during opportunity discovery, grouped into thematic areas.

Code	Thematic area^a^	Example needs statements^b^
MI	Medical information (n=14)	“Doctors need a way to get accurate medical information (signs and symptoms) about the patient in order to provide correct diagnosis and treatment plan” [MI.01] “FHWs need a way to accurately record patients’ medical information (signs and symptoms) and share it with the remote doctor to improve the quality of diagnosis and treatment.” [MI.02]
KC	Frontline health worker knowledge and competency (n=14)	“Health organizations need to standardize the skills of FHWs so that all kiosks can provide quality care.” [KC.12]
ID	Instrumentation and diagnostics (n=9)	“Doctors need high quality, accurate stethoscope results (heart and lung sounds) to effectively diagnose patients.” [ID.04]
MC	Medications and compliance (n=4)	“Doctors and FHWs need a way to improve patient compliance with medical advice and medications to improve patient outcomes.” [MC.01]
IC	Patient education and informed consent (n=4)	“FHWs need a way to explain concepts of informed consent (such as risks and benefits of telemedicine and data privacy) in a manner that is comprehensible to rural patients with low literacy backgrounds so that patients can make informed decisions.” [IC.02]
PX	Patient experience (n=4)	“Patients need to feel like their information is accurately conveyed to the Doctors to engender trust in the kiosk model and increase patient acceptance.” [PX.04]
EC	Emergency care (n=4)	“Health organization, FHWs, and doctors need to better identify patients needing emergency services to provide first aid, stabilize the patient, and promptly initiate a referral.” [EC.01]
CW	Clinical workflows (n=6)	“Health organizations need to optimize patient flow, reduce process redundancies, and increase patient throughput to improve teleconsultation efficiency.” [CW.01]
CO	Communication (n=5)	“Patients, FHWs, and doctors need to communicate in a language that is comfortable for all stakeholders in the telemedicine interaction.” [CO.01]
TE	Telecommunications infrastructure (n=3)	“Health organizations, FHWs, and doctors need technology to function reliably, including during power outages and periods of low/no internet.” [TE.03]
EU	Ease of use (n=2)	“Doctors and FHWs need technology which does not consume a lot of time in data entry and fits seamlessly into the clinical workflows.” [EU.02]
FS	Financial sustainability (n=5)	“Health organizations need to have a very high operational efficiency to achieve sustainability and scalability of the model.” [FS.01]

^a^The number in parentheses includes the total number of needs statements in that thematic area. For a complete list, see Multimedia Appendix 2.

^b^For the complete list of example needs statements (a total of 74), see Multimedia Appendix 2.

### Iteration 2: Needs Selection

#### Task Shifting in Telemedicine

Through interviews with the remote physicians, we recognized that there was a limitation on the types of patients for which a management plan could be remotely developed, owing mainly to the lack of trustworthy elicitation of the patients’ signs and symptoms. The accurate collection of medical information and its communication between the various stakeholders is essential to arrive at the correct diagnosis and treatment plan for the patient. Overall, the more relevant data the FHW can reliably collect from the patient via history taking, physical examinations, and point-of-care diagnostic sensors, the higher the ability of the remote physician to diagnose accurately. After evaluating each need on the 4 quadrants using the evaluation parameters (Multimedia Appendix 1), we identified the need for task shifting clinical information gathering to an FHW as a top need (MI.01; Table 1).

#### Significance of Task Shifting Information Gathering to an FHW in a Telemedicine Setting

In telemedicine, the remote physician is not in the same location and cannot directly see, touch, or hear the patient. The FHW serves as the “ears, eyes, and hands” of the physician [11]. The FHW does not have the required training or skill to be able to collect this information accurately. Collecting a comprehensive medical history and performing a clinical examination, the main pillars of arriving at a provisional diagnosis, require medical knowledge, training, and experience. The FHW typically cannot be trained comprehensively in these skills (short of going through medical school and a residency program). Hence, most telemedicine programs involve the FHW simply registering the patient with basic demographic details, serving as a telecommunications operator, and the remote physician does the remaining patient interview processes over the phone or a video call.

Task shifting history taking to other types of health workers or directly to the patient for self-reporting with the use of a digital assistant or a computer-assisted history-taking system to improve the quality of clinical information gathering has been well established in the literature, mainly being used in health care settings in high-income, developed countries [12,13]. A review of the literature reveals some clear benefits such as the improvement in documentation, reduction in time spent by a provider in documentation, the ability to collect more comprehensive and relevant information, and improvement in the quality of information gathered [12]. The drawbacks include the inability to capture nonverbal communication, frustration felt by users if the questions do not fit the scenario, user interface challenges, and irrelevant questioning [12].

It is essential to understand the role of patient history, physical examinations, and investigations in arriving at a diagnosis. A study in India with 100 in-person outpatient consultations showed that in 78.58% of the cases, the patient history led to the diagnosis [17]. In 8.17% of patients, the physical examinations led to a diagnosis, and in 13.27% of patients, investigations led to a diagnosis [17]. The study also showed that the physician’s confidence in the correct diagnosis increased subsequently at each stage of the clinical information–gathering process from 6.36 after history taking to 7.57 after physical examinations and to 9.87 after investigations as measured on a Likert scale from 1-10 [17].

A retrospective analysis of 32 malpractice suits in telephone consultation–related adverse events showed that poor documentation (88% of the 32 cases) and faulty triage decisions because of incomplete history taking over the phone (84% of the 32 cases) were the leading causes of diagnostic error [24]. A study by Resneck et al [25] observed history taking to be rushed or incomplete in direct-to-patient web-based consultations because of time pressures as well as the physician not being able to see the patient. Simple relevant questions related to history taking, including allergies, medications, or pregnancy status, were routinely missed, resulting in missed diagnoses [25].

In rural India, access to diagnostic laboratories is poor, necessitating additional patient travel to conduct basic laboratory tests. We observed that the physician often has to rely on a well-taken history to arrive at a diagnosis and management plan. This places added value on the patient interview because it is often the primary basis for decision-making.

Although the patient interview is an essential component of the diagnostic decision-making process, history taking in resource-limited settings may often be rushed or incomplete because physicians are overburdened. A systematic review of the average time taken for a primary care consult in 71 countries showed a wide variation in the average time taken for a consult between developed and developing countries [16]. For example, an average primary care consult in India lasts 2.5 minutes and an average primary care consult in Bangladesh lasts 48 seconds [16].

A study in rural India used standardized patient actors to assess the quality of care of health care delivery by 224 public sector and private sector providers (qualified and unqualified). It analyzed the care quality in terms of consultation length. In addition, the study used a checklist of essential history taking and examination steps that providers should follow during a consultation and evaluated what percentage of these steps were actually followed by the providers. The average public sector provider-patient interaction lasted 2.4 minutes during which the provider completed 16% of the checklist items, and the average private sector provider-patient interaction lasted 3.7 minutes and gathered 22% of information from the essential checklist [26]. The consultation length was strongly correlated with the completion of more items on the checklist [26]. We observed similar concerns through our ethnographic research. The time taken by the physician to complete a teleconsultation is often a function of the patient load, which is very high in resource-limited settings. Optimizing the amount of time spent by a physician on a teleconsultation, without compromising the quality of care, has important implications for the financial sustainability and scalability of telemedicine in LMICs.

### Iteration 3: Solution Design and Development

#### Overview

**Figure 1:**
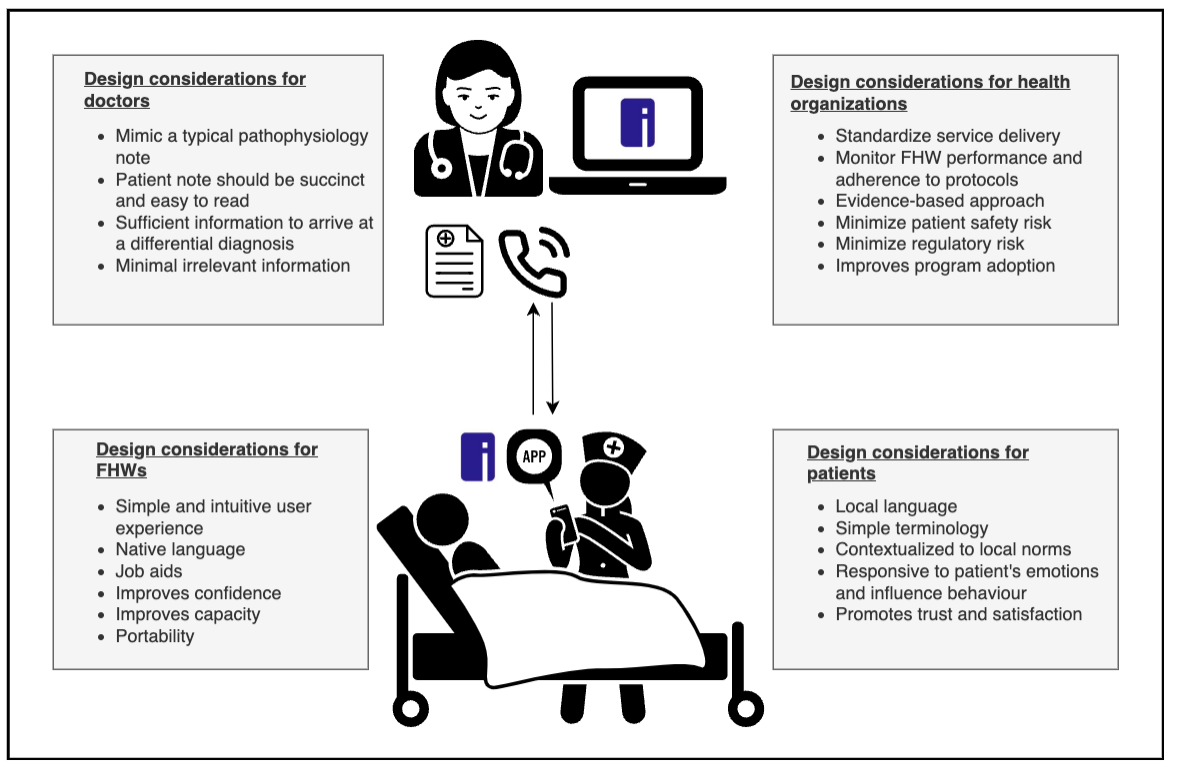
Schematic summarizing the key design considerations to be addressed by the solution. FHW: frontline health worker.

We integrated perspectives from all 4 quadrants when designing the prototype. We developed multiple versions of the prototype and refined them. Insights from other needs statements were also incorporated into the design so that the overall solution could address several unmet needs. These are summarized in Figure 1.

The final prototype was a “digital assistant” that enables the task shifting of history taking and physical examination tasks to FHWs to allow for effective teleconsultations. The goal of this assistant was not to provide a final diagnosis but to guide the FHWs to collect comprehensive patient information to share with the remote physician.

#### Workflow

The FHW uses a mobile app with the digital assistant for elucidating clinical information from patients and also conducts some diagnostic tests using point-of-care diagnostic devices (blood pressure, blood sugar, electrocardiogram, etc). The combined data are sent to a remote physician using low bandwidth internet for evaluation and decision on the clinical management pathway. The physician, on reviewing the case, communicates with the FHW and the patient over a phone call or a video call for further clarification or examination and accordingly prescribes medications and provides advice. This information is then transmitted to the FHW who prints the prescription, explains, and hands it over to the patient. Patients can purchase some basic medicines from a pharmacy nearby. Emergency patients or patients who cannot be managed via telemedicine are referred to the nearest secondary or tertiary care facility. The workflow of a teleconsultation when guided by the digital assistant is shown in Multimedia Appendix 3.

#### Mobile App

We developed the task-shifting tool as a mobile app because of the growing use of low-cost mobile devices in global health care delivery. Medical history taking and physical examination are core clinical skills taught to physicians and often require years to attain proficiency. As FHWs do not have the medical knowledge and training to take a complete, evidence-based medical history or to conduct examinations as a qualified physician would, a mix of job aids was built into the tool to guide them contextually in what to ask and what to examine. We also developed a training protocol for the use of the tool. We developed the interface keeping in mind the need to promote improved confidence and increased capacity in FHWs to execute these skills at an adequate level of proficiency.

A sense of trust between the physician and the health worker is essential to their functioning effectively together as a care team. The fact that an evidence-based, knowledge-enabled digital tool to facilitate task shifting in a high-quality, standardized manner is behind the FHWs’ workflow can increase this trust while enhancing the quality of the information. The output note shared with the physician was concise and easy to read to enable efficient communication. It aimed to have all the information that is necessary and sufficient to arrive at a differential diagnosis. Irrelevant information that serves as “noise” for the physician, making it harder to focus on the information relevant to the case, was minimized.

The primary language of communication among the patient, FHW, and physician is the local language. This placed additional considerations on the use of terminology and proper translations of medical terms into the local language (Bengali). Medical terms were presented in a simple language that was easy for both the FHW and the patient to comprehend. History-taking questions were contextualized to local norms. We hypothesized that more time would be spent on history taking and examination when conducted by an FHW with the digital assistant instead of the 2 to 3 minutes spent by a busy physician in an overcrowded outpatient clinic. This would have a profound impact on improving the patient experience. Overall, the solution was designed keeping in mind the need to meet the sociological and psychological goals of the patient interview, that is, responding to patients’ emotions and influencing their behavior. Data collected by the mobile app are stored in an open-source EMR system (OpenMRS) to ensure that each patient’s clinical information is tracked longitudinally and tied to a unique health identifier.

#### Protocol Development: Defining What Should Be Task Shifted and What Can Be Task Shifted

While building the protocols, it was pertinent to determine what could be safely and effectively task shifted to FHWs:

- Should abdominal palpation to elicit superficial tenderness be task shifted to an FHW?
- Should the collection of sexual history be task shifted to an FHW?
- Should the measurement of blood pressure be task shifted to an FHW?

The decision about what to task shift depends on the current education and skill level of the FHW; the amount of time and complexity required to train them in new skills; their ability to retain those new skills; and the feasibility of executing continuous training, learning, and competency assessment. The return on investment on training the FHW versus the value of the specific information to make a diagnosis given the context drives the decision of whether a specific skill should be task shifted. The patient’s willingness to share this information with the FHW when asked is an important consideration for deciding whether it can be task shifted. Thus, the need to collect specific data should be balanced with the acceptability and trainability of the health worker to gather it accurately.

#### Clinical Value of the Symptom or Sign in Making a Diagnosis

**Figure 2:**
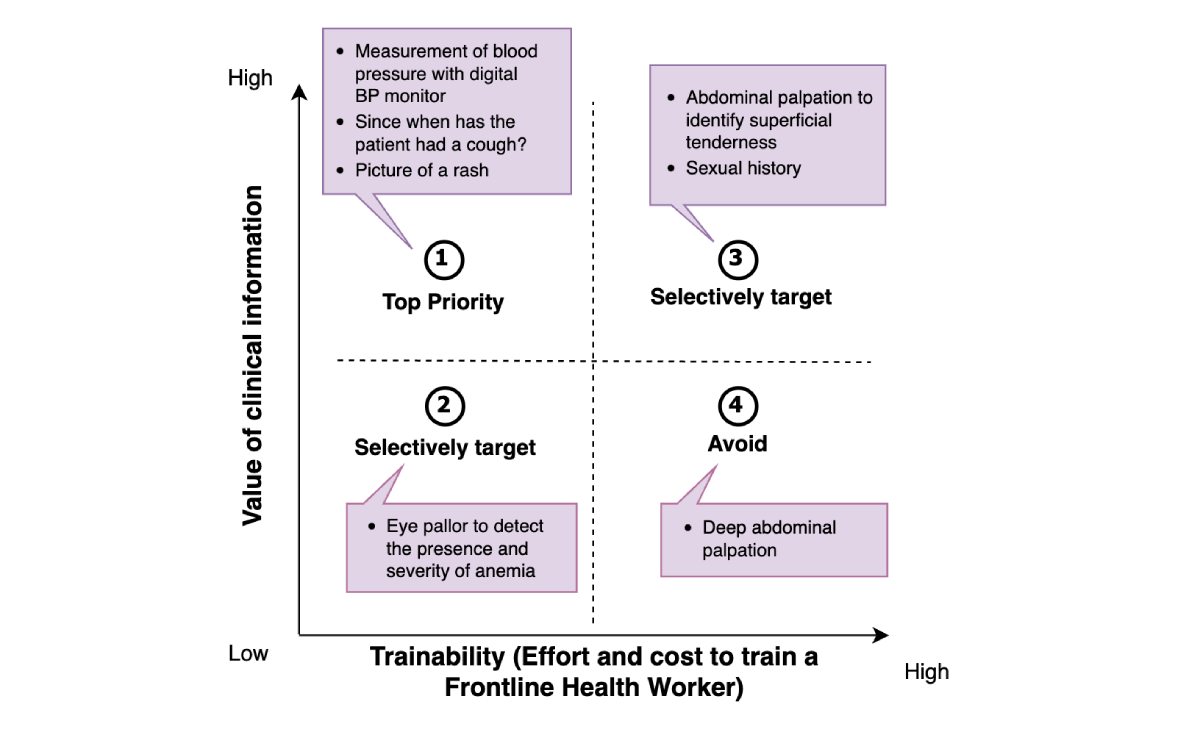
Framework for prioritizing what signs and symptoms are included in the task-shifting digital assistant with examples. We selected clinical information that is of high value and low effort to train a frontline health worker (FHW), with high patient and FHW acceptability as suitable targets for task shifting. Both the trainability and the clinical value are dependent on the baseline skill level of the FHW (nurse vs midwife vs community health worker) and the socioeconomic context of the clinic, such as its distance from the nearest diagnostic center. BP: blood pressure.

Key symptoms and signs allow physicians to rule in or rule out a diagnosis. The value of a symptom or sign is usually thought of in the context of making a diagnosis. The patient interview progresses in such a way as to arrive at a differential diagnosis. After this, the physician can order further tests to confirm a diagnosis or pursue a therapeutic pathway by making the best decision from the available data. Red flag symptoms that rule in diagnoses that would result in death or severe disability are “high value” because they allow the remote physician to identify patients needing urgent care or referral. Thus, the clinical value of a symptom or sign is intrinsically related to the morbidity of the diagnosis it can point to, its sensitivity, specificity, negative and positive predictive value, and its contribution toward deciding the appropriate therapeutic plan (Figure 2).

#### Level of Effort Needed to Train the FHW in Capturing the Symptom or Sign

Task shifting of data gathering for each sign or symptom is associated with a level of training that needs to be provided so that the FHW has the skills required to collect it accurately. For example, asking a patient since when they have had back pain and then selecting the duration of the pain does not require a lot of training, especially if the tool prompts the question and the FHW does not have to remember the context in which the question needs to be asked. Measuring vital signs using point-of-care devices requires a higher level of training. Examinations such as chest auscultation or palpating the margins of the liver require an extremely high burden of training and practical experience (Figure 2).

#### Acceptability to the Patient

From direct observations of multiple client-provider health interactions, we noticed that collecting information about sexual history for men by female health workers or questions about mental health issues may not be considered culturally appropriate for an FHW to probe as they are members of the same community. Furthermore, we hypothesized that acceptability relates to several aspects of the FHW—their gender and social status relative to the patient, the perception of their expertise in the eyes of the patient, and the prevalent sociocultural norms.

#### Acceptability to the Health Worker

We also observed that task shifting creates an additional workload for the health workers. Furthermore, FHWs in LMICs are often not well-compensated and are overburdened, leaving little incentive to take on other tasks. We observed that the health worker’s confidence in being able to use a digital tool or devices can also limit acceptability.

#### Regulatory Considerations

From the health organization’s perspective, such a tool would allow for standardization in task shifting so that FHWs with varying skills and abilities could perform consistently at high quality. An important consideration for health organizations is in assuming the regulatory uncertainty around task shifting to FHWs because the guidelines are often not laid out. An evidence-based approach to the development of protocols was adopted to minimize patient safety risks and minimize regulatory risks. Furthermore, although data and interoperability standards are currently in a nascent stage in India, as government regulations and standards for telehealth systems are adopted, the tool would also need to adhere to these standards to achieve integration with other similar vertical systems and prevent data from being siloed. Hence, we chose to use OpenMRS as an EMR backend for the system so that interoperability requirements could be achieved via the OpenMRS Fast Healthcare Interoperability Resources, Health Level 7 APIs, and the use of data dictionaries such as Systematized Nomenclature of Medicine Clinical Terms, Logical Observation Identifiers Names and Codes, and International Classification of Diseases, ninth and tenth revisions, already built into the OpenMRS platform architecture [27].

We built a committee of 11 members across all the stakeholder groups (distinct from the stakeholders observed in phase 1) to develop and review the protocols. Task-shifting protocols were developed from known evidence bases such as medical textbooks or guidelines issued by the health ministry with clear citations to the source of the protocol. Different members of the committee participated in the development as it related to their background and expertise (Table 2).

**Table 2:** Process of knowledge acquisition to develop task-shifting protocols to collect patient information^a^.

Step	Stage	Result	Committee members involved				
			1: physician	2: physician	3: physician	4 and 5: program manager	6, 7, 8, 9, 10, and 11: community health workers
Step 1	Identify symptom list to cover the scope of most prevalent presenting complaints through literature review	67 presenting complaints identified	✓	✓	✓		
Step 2	Create data collection questionnaires to collect subjective data for the presenting complaints through a literature review and synthesis of evidence-based guidelines	67 data collection questionnaires compiled	✓	✓	✓		
Step 3	Identify simple physical examinations to collect objective data and map them to complaints	143 examinations identified	✓	✓	✓		
Step 4	Contextualization of questionnaires to the etiology and epidemiology of disease in India	67 questionnaires contextualized		✓	✓	✓	
Step 5	Feasibility assessment to remove history-taking questions and physical examinations that are difficult to task shift to health workers or have a high burden of training	Questionnaire list reduced to 51; examination list reduced to 93		✓	✓	✓	
Step 6	Translation of content into local language (Bengali) and adaptation to improve comprehensibility for patients	Translations complete and verified; 51 questionnaires and 93 physical examinations modified			✓	✓	✓
Step 7	Adaptations to local social and cultural contexts	Adaptations complete and verified; 51 questionnaires and 93 physical examinations modified				✓	✓

^a^Physicians were responsible for the curation of medical knowledge to build the protocols. Public health program managers were experts in working with community health workers as well as in representing patient needs. The community health workers represented the sociocultural norms of the community that they served and the local dialect as well as patient needs.

### User Feedback Interviews

The first version of the prototype was shared with the health workers, physicians, and public health program managers who were observed in the ethnographic design stage for feedback. The users saw a demo of the tool and directly interacted with the app for 1 hour. All the participants agreed that the digital assistant would be a useful addition to the telemedicine program and improve key project bottlenecks. The physicians and the public health program managers felt that task shifting the patient interview to an FHW may save the physician’s time and increase their ability to diagnose remotely. It could lead to better documentation of consults and increase the availability of actionable data for public health analysis. A physician observed that structured information in the output note can be used to trigger physician job aids such as differential diagnosis checklists and standard treatment guidelines.

Community health workers felt that the tool could improve their ability to interact with the patient and reduce back and forth communication with physicians. It would also increase the patient’s trust in their skills and in the program overall. They were concerned about whether they would be able to use the tool correctly and provided feedback to improve the user interface and make it simpler. All users expressed that the community health workers would need to be trained properly to use the tool effectively. Some users expressed concern that this may make the patient encounter too lengthy because of the detailed nature of the questioning workflows. The public health program manager observed that such a tool also can potentially implement further evidence-based medicine approaches to improve clinical care delivery in health worker–to-physician telemedicine programs. Owing to the small sample size, these findings cannot be generalized.

**Figure 3:**
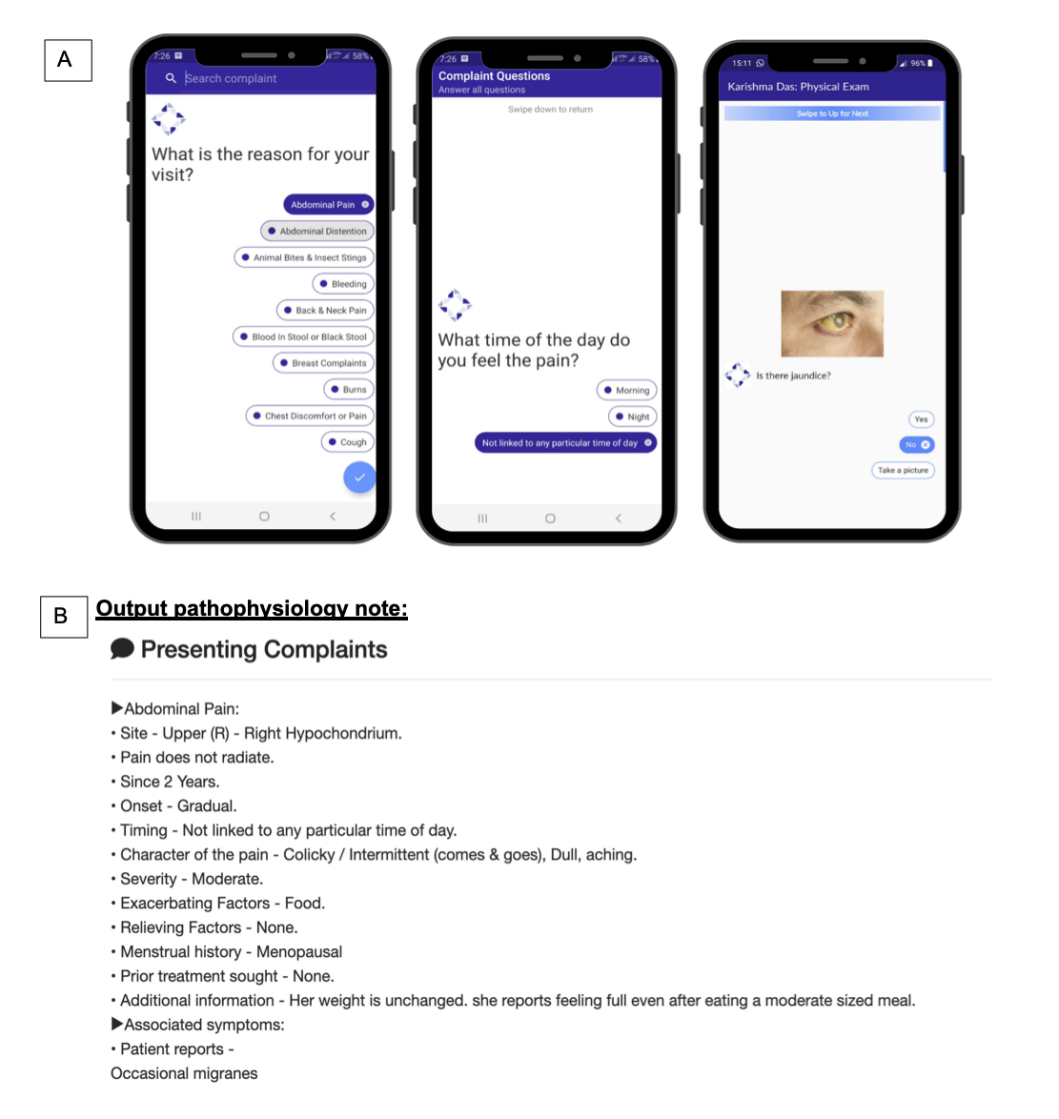
(A) The user interface of the prototype digital assistant. The frontline health worker (FHW) can select the presenting complaints, answer detailed questions for each complaint, and collect past medical history and family history using a standard protocol–based adaptive questionnaire. The FHW collects vital signs and is guided through conducting physical examinations. (B) An example output note generated by the FHW using the digital assistant. The assistant also guides the FHW to capture images to share with the physician so that the physician may arrive at a diagnosis.

The first prototype thus addressed several needs identified in the opportunity discovery phase and gave us insight into concerns. Accordingly, the final version of the app that was developed incorporating the user feedback has been shown in Figure 3.

We developed the prototype version with history-taking questionnaires for 51 presenting complaints and 93 physical examinations (Figures 3A and 3B). We released the app source code under the free and opensource Mozilla Public License 2.0 [28]. Supporting documentation is provided on the internet [29].

### Limitations and Strengths

Many of these conclusions are based on a design ethnography process. A different set of user needs may have been discovered by a different set of researchers. The results are derived from observations in 2 communities in a single state in India. One has to be cautious in interpreting the generalizability of this approach to multiple geographies. Although the participants reported both positive and negative feedback about the usefulness of the digital assistant, there is a high chance of positive bias in the user feedback interviews. The responses cannot be generalized because of the small sample size. Further validation of these hypotheses is required to see if these needs and the resulting solution can be applied broadly.

Although many digital assistants for history taking have been developed in high-resource settings, to the best of our knowledge, no such system has been designed comprehensively with the needs and requirements of a rural health worker–to-physician community-based telemedicine program in a resource-constrained environment.

### Conclusions

A digital tool for task shifting clinical information gathering to an FHW has high significance and value in a telemedicine setting in an LMIC as observed through interviews with key stakeholders. We identified the key value propositions and user needs and presented a prototype of a task-shifting tool for telemedicine settings in LMICs. In a developing country setting, such a tool’s significance may be much higher, given the resource constraints that physicians operate under. The final prototype incorporated unique value propositions for all stakeholders—physicians, FHWs, patients, and public health program managers—and could result in an overall improvement in the quality of care delivered via telemedicine in resource-constrained environments. The prototype version was acceptable to the users. Future scope for development of this tool would involve additional iterations of the spiral innovation approach with the refinement of the prototype, testing, regulatory compliance, pilot implementation, field evaluation, and commercial validation. Additional research needs to be conducted to evaluate the digital assistant and its impact on various aspects of a telemedicine program such as its feasibility of implementation, impact on the diagnostic outcome, impact on improving health worker capacity and competence, patient and provider satisfaction, clinical safety, program quality, and efficiency of care delivery.

## Appendix

Multimedia Appendix 1 The Spiral-Iterative Innovation Model that guided the 3 phases of (A) opportunity discovery, (B) needs selection, and (C) solution design.

Multimedia Appendix 2 Needs statements identified during opportunity discovery, grouped into thematic areas.

Multimedia Appendix 3 Swimlane diagram with the workflow of a patient–health worker–physician teleconsultation guided by a task-shifting tool.

## Abbreviations

EMR: electronic medical record
FHW: frontline health worker
HA: health assistant
LMIC: low- and middle-income country
