# Supplementary figures and images for: Development of a Digital Assistant to Support Teleconsultations Between Remote Physicians and Frontline Health Workers in India: User-Centered Design Approach
Source: JMIR Hum Factors. 2023 Feb 2;10:e25361. doi: 10.2196/25361 (PMC9936362; doi:10.2196/25361)

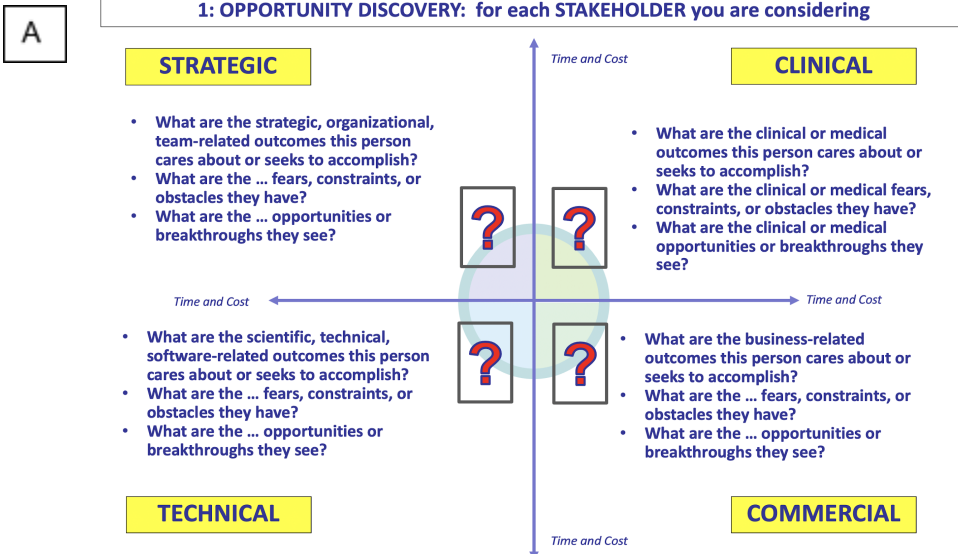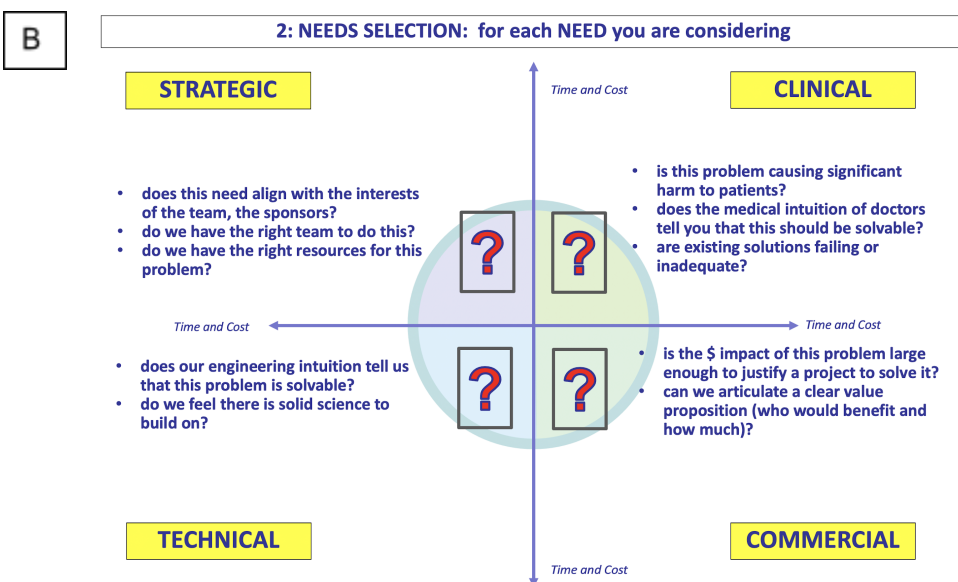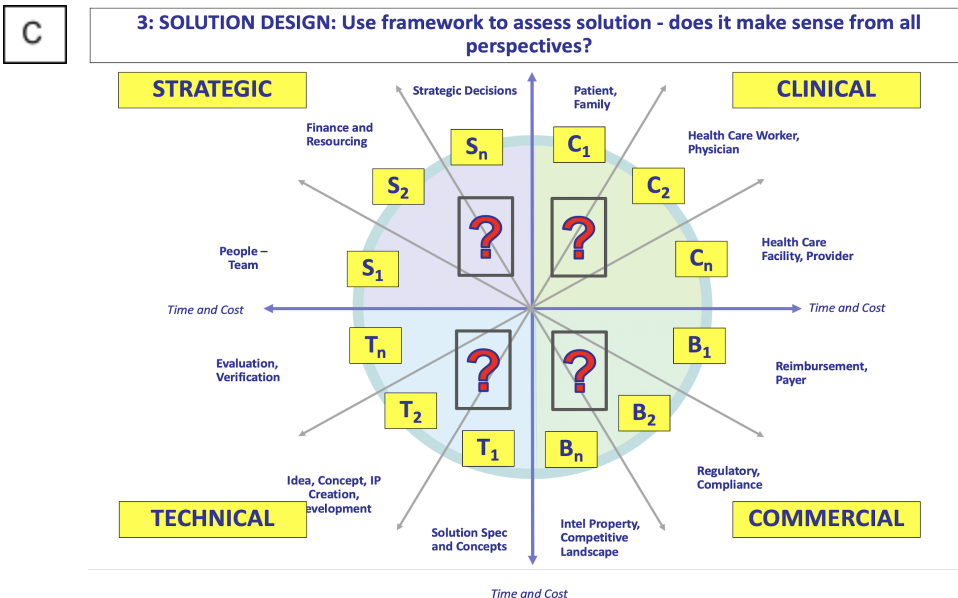

Supplement: Multimedia Appendix 1 [file humanfactors_v10i1e25361_app1.pdf]

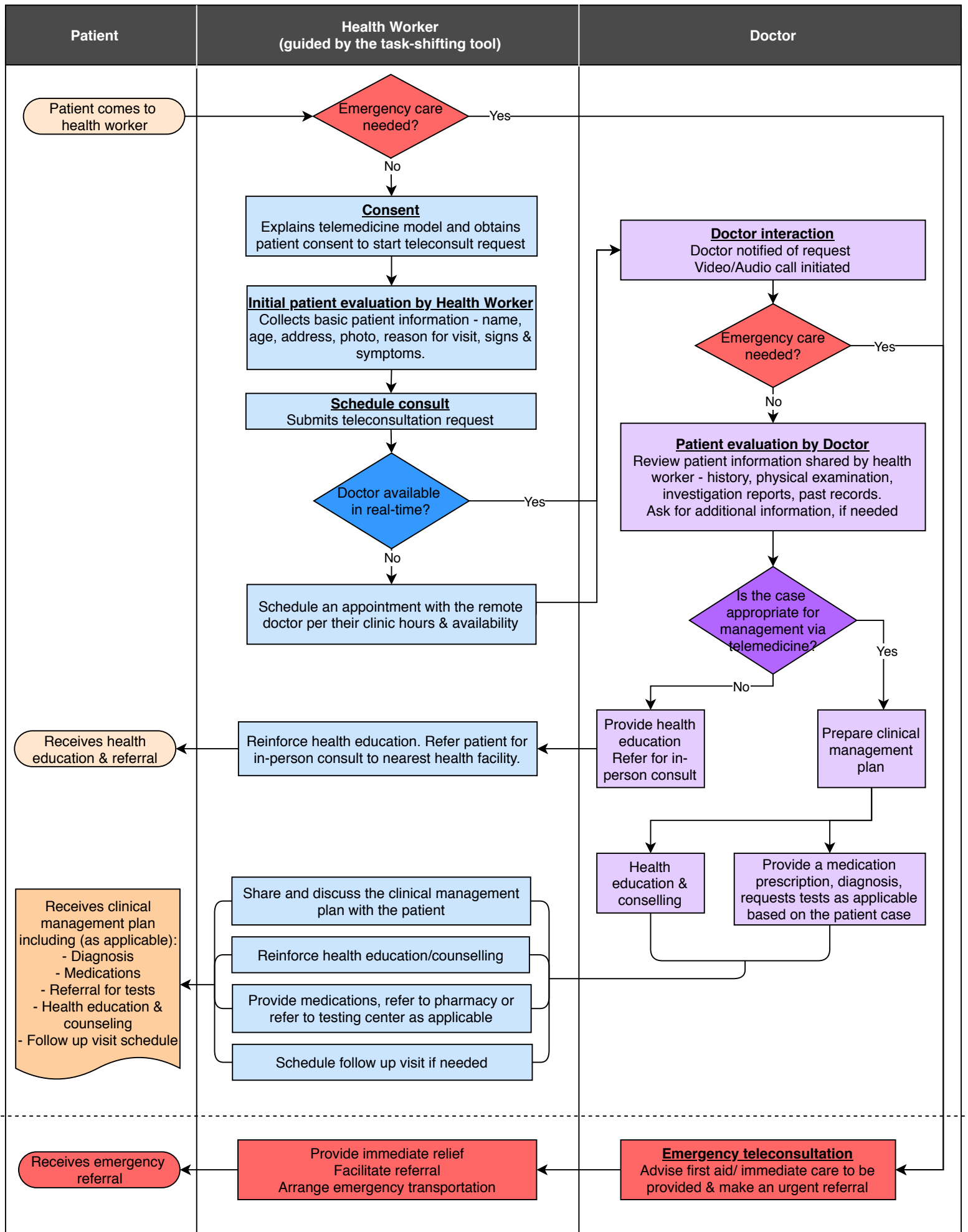

Supplement: Multimedia Appendix 3 [file humanfactors_v10i1e25361_app3.pdf]
