# Supplementary material for: Development of a Digital Assistant to Support Teleconsultations Between Remote Physicians and Frontline Health Workers in India: User-Centered Design Approach
Source: JMIR Hum Factors. 2023 Feb 2;10:e25361. doi: 10.2196/25361 (PMC9936362; doi:10.2196/25361)
Supplement: Multimedia Appendix 2 [file humanfactors_v10i1e25361_app2.docx]

**Needs statements identified during opportunity discovery, grouped into thematic areas**

| NO. | CODE | THEMATIC AREA | NEED STATEMENT |
| --- | --- | --- | --- |
| 1 | MI.01 | Medical information | Doctors need a way to get accurate medical information (signs & symptoms) about the Patient in order to provide correct diagnosis and treatment plan |
| 2 | MI.02 | Medical information | FHWs need a way to accurately record Patient's medical information (signs & symptoms) and share it with the remote Doctor in order to improve the quality of diagnosis and treatment |
| 3 | MI.03 | Medical information | FHWs need to know which physical exams should be performed for the patient in order to share the necessary clinical information with the Doctor |
| 4 | MI.04 | Medical information | FHWs need a way to reduce data entry and spelling errors in order to improve quality and legitimacy of clinical information |
| 5 | MI.05 | Medical information | FHWs need to improve the quality of medical information (signs & symptoms) that they collect in order to inspire Doctor and Patient confidence |
| 6 | MI.06 | Medical information | FHWs need a way to better convey the severity of Patient's illness to the Doctor in order to improve quality of diagnosis and treatment |
| 7 | MI.07 | Medical information | FHWs need a way to obtain and share Patient's labratory results with the Doctor in order to improve quality of diagnosis and treatment |
| 8 | MI.08 | Medical information | FHWs need a way to send visual information (such as a picture of a rash) to Doctors in order to improve quality of diagnosis and treatment |
| 9 | MI.09 | Medical information | FHWs need a way to provide the Doctor's prescription & treatment plan to the Patient |
| 10 | MI.10 | Medical information | Patients need a simple way to access and keep track of their medical information |
| 11 | MI.11 | Medical information | Doctors need a way to keep track of Patient's medical information in order to pull up previous visit notes/diagnoses/lab results and improve quality of diagnosis and care |
| 12 | MI.12 | Medical information | FHWs need a way to keep track of Patient's medical information in order to pull up previous visit notes/diagnoses/lab results and improve quality of diagnosis and care |
| 13 | MI.13 | Medical information | Doctors need a way to ensure that their advice and prescription are not being modified by the FHW in order to be comfortable with prescribing remotely |
| 14 | MI.14 | Medical information | FHWs need a reliable way to send high quality visual information to Doctors in order to enable accurate dermatological diagnosis |
| 15 | KC.01 | FHW Knowledge & Competency | FHWs need to fully understand the extent of their role vs that of the Doctor in order to ensure patient safety & comply with regulatory guidelines of what FHWs are allowed to provide care for |
| 16 | KC.02 | FHW Knowledge & Competency | FHWs need a way to learn more clinical skills in order to improve the number of health services that can be provided at the kiosk |
| 17 | KC.03 | FHW Knowledge & Competency | FHWs need a safe and effective way to practice their clinical skills in order to improve quality of care |
| 18 | KC.04 | FHW Knowledge & Competency | FHWs need to a way to continuously improve their competency in order to provide better quality care |
| 19 | KC.05 | FHW Knowledge & Competency | FHWs need a way to improve their healthcare knowledge in order to improve quality of care |
| 20 | KC.06 | FHW Knowledge & Competency | FHWs need to improve their bedside manner in order to provide a positive patient experience |
| 21 | KC.07 | FHW Knowledge & Competency | FHWs need to improve their confidence in in their skills in order to inspire patient confidence |
| 22 | KC.08 | FHW Knowledge & Competency | FHWs need a way to better learn new technology applications in order to reduce their fear of and incidence of errors |
| 23 | KC.09 | FHW Knowledge & Competency | FHWs need to be more familiar with the technology so they can give more attention to the patient |
| 24 | KC.10 | FHW Knowledge & Competency | FHWs need an easy and effective way to practice their technology skills |
| 25 | KC.11 | FHW Knowledge & Competency | FHWs need to understand the technology better so they can troubleshoot small issues |
| 26 | KC.12 | FHW Knowledge & Competency | Health organization needs to standardize the skills of FHWs so that all kiosks can provide quality care |
| 27 | KC.13 | FHW Knowledge & Competency | Health organization needs to standardize the skills of FHWs so that Doctors can reliably trust the quality of medical information |
| 28 | KC.14 | FHW Knowledge & Competency | Health organization needs a way to ensure FHW safety |
| 29 | ID.01 | Instrumentation/ Diagnostics | Patients need to receive a wide range of diagnostic tests without having to travel long distances in order to improve the quality of care |
| 30 | ID.02 | Instrumentation/ Diagnostics | FHWs need a way to accurately measure vital signs in order to provide quality information to the doctors |
| 31 | ID.03 | Instrumentation/ Diagnostics | FHWs need a way to accurately measure patient blood pressure in order to provide quality information to the doctors and allow for accurate hypertension monitoring |
| 32 | ID.04 | Instrumentation/ Diagnostics | Doctors need high quality, accurate stethoscope results (heart and lung sounds) in order to effectively diagnose patients |
| 33 | ID.05 | Instrumentation/ Diagnostics | Patients need an easier way to monitor their blood sugar levels in order to adjust their diet and reduce risk of diabetic complications |
| 34 | ID.06 | Instrumentation/ Diagnostics | Doctors need a quick way to know/assess results of the ECG test in order to provide high quality diagnosis and any needed referral/advice |
| 35 | ID.07 | Instrumentation/ Diagnostics | Doctors and FHWs need a better way to provide treatment for patient muscle and joint pain in order to improve patient treatment |
| 36 | ID.08 | Instrumentation/ Diagnostics | Health organization needs highly durable equipment in order to reduce costs associated with replacing broken technologies |
| 37 | ID.09 | Instrumentation/ Diagnostics | Patients need a better way to monitor sugar intake in order to reduce their risk of diabetic complications |
| 38 | MC.01 | Medications and compliance | Doctors and FHWs need a way to improve Patient compliance to medical advice and medications in order to better improve patient outcomes |
| 39 | MC.02 | Medications and compliance | Doctors need a way to evaluate/know level of Patient compliance in order to assess if the prescribed drug was ineffective and should be changed to improve patient health outcomes |
| 40 | MC.03 | Medications and compliance | Patients need to receive prescriptions with generic/affordable medications in order to improve affordability of medicines and improve compliance |
| 41 | MC.04 | Medications and compliance | Patients need to receive good quality affordable medications from pharmacies so they can receive needed care and be confident in the kiosk |
| 42 | IC.01 | Patient education & Informed consent | Health organization, FHWs and Doctors need to better communicate to Patients the range of care provided by the kiosk and the limits of telemedicine |
| 43 | IC.02 | Patient education & Informed consent | FHWs need a way to explain concepts of informed consent (such as risks and benefits of telemedicine and data privacy) in a manner that is comprehensible to rural patients with low literacy backgrounds so that patient's can make informed decisions |
| 44 | IC.03 | Patient education & Informed consent | Doctors and FHWs need a better way to explain chronic disease and health risk to Patients in order to improve compliance and patient health outcomes |
| 45 | IC.04 | Patient education & Informed consent | Patients need access to preventative health information and knowledge |
| 46 | PX.01 | Patient experience | Patients need to feel like they are being treated with dignity and respect |
| 47 | PX.02 | Patient experience | Health organization needs to ensure that the kiosk is open at a convenient time for Patients in order to improve access to care |
| 48 | PX.03 | Patient experience | Patients need to feel like they are getting quality healthcare in order to ensure compliance and increase rate of returning Patients |
| 49 | PX.04 | Patient experience | Patients need to feel like their information is getting accurately conveyed to the Doctors in order to engender trust in the kiosk model and increase patient acceptance |
| 50 | EC.01 | Emergency care | Health organization, FHWs and Doctors need to better identify Patients needing emergency services in order to provide first aid, stabilize the patient and promptly initiate a referral |
| 51 | EC.02 | Emergency care | FHWs and Doctors need a way to communicate with patients that emergency cases cannot be managed over telemedicine and that urgent referral is required |
| 52 | EC.03 | Emergency care | Patients need a faster way to travel to hospitals for emergency cases in order to improve health outcomes |
| 53 | EC.04 | Emergency care | FHWs need a way to help Patients needing emergency care to reach the nearest hospital |
| 54 | CW.01 | Clinical workflows | Health organizations need to optimize patient flow, reduce process redundancies and increase patient throughput in order to improve teleconsultation efficiency |
| 55 | CW.02 | Clinical workflows | FHWs need to improve the efficiency of patient intake in order to optimize patient throughput and become financially sustainable |
| 56 | CW.03 | Clinical workflows | FHWs need a fast and accurate way to collect and record medical information in order to improve teleconsultation efficiency |
| 57 | CW.04 | Clinical workflows | Doctors need a fast and accurate way to collect and record medical information about the patient in order to improve teleconsultation efficiency |
| 58 | CW.05 | Clinical workflows | FHWs need a way to easily remind patients about follow-up visits in order to ensure quality patient care |
| 59 | CW.06 | Clinical workflows | Health organization needs a way to better schedule Doctor and Patient interactions order to improve efficiency and improve patient satisfaction |
| 60 | CO.01 | Communication | Patients, FHWs and Doctors need to communicate in a language that is comfortable for all stakeholders in the telemedicine interaction |
| 61 | CO.02 | Communication | FHWs need an easy way to explain the telemedicine model to Patients in order to improve patient acceptability |
| 62 | CO.03 | Communication | FHWs need a way to convey accurate information to the Doctor when the internet is not available |
| 63 | CO.04 | Communication | Doctors need a way to convey medical advice and prescriptions to the FHW & Patient when the internet is not available |
| 64 | CO.05 | Communication | FHWs need tools to communicate and reinforce the doctor's advice to the Patient |
| 65 | TE.01 | Telecommunications infrastructure | FHWs need a more reliable way to transfer Patient information and data to Doctors over low bandwidth internet |
| 66 | TE.02 | Telecommunications infrastructure | Health organizations need to ensure that there is reliable internet and power at the kiosk in order to function effectively |
| 67 | TE.03 | Telecommunications infrastructure | Health organizations, FHWs and Doctors need technology to function reliably, including during power outages and periods of low/no internet |
| 68 | EU.01 | Ease of use | Doctors and FHWs need technology that is easy to use in order to ensure user adoption |
| 69 | EU.02 | Ease of use | Doctors and FHWs need technology which does not consume a lot of time in data entry and fits seamlessly into the clinical workflows |
| 70 | FS.01 | Financial sustainability | Health organization need to have a very high operational efficiency in order to achieve sustainability and scalability of the model |
| 71 | FS.02 | Financial sustainability | Health organization needs to price health services appropriately in order to balance accessibility of care with profitability of the kiosk |
| 72 | FS.03 | Financial sustainability | Health organizations need to minimize churn of FHWs |
| 73 | FS.04 | Financial sustainability | Health organization needs a way to task shift routine follow up care/monitoring of chronic care patients to FHWs in order to reduce unnessary doctor time and improve financial sustainability |
| 74 | FS.05 | Financial sustainability | Health organization needs to ensure that patients are recieving high quality care in order to improve patient retention and kiosk sustainability |
